# Role of MicroRNA in Proliferation Phase of Wound Healing

**DOI:** 10.3389/fgene.2018.00038

**Published:** 2018-02-14

**Authors:** Amro M. Soliman, Srijit Das, Norzana Abd Ghafar, Seong Lin Teoh

**Affiliations:** Department of Anatomy, Universiti Kebangsaan Malaysia Medical Centre, Kuala Lumpur, Malaysia

**Keywords:** angiogenesis, chronic wound, healing, microRNA, re-epithelialization

## Abstract

Wound healing is a complex biological process that is generally composed of four phases: hemostasis, inflammation, proliferation, and remodeling. The proliferation phase is crucial for effective healing compared to other phases. Many critical events occur during this phase, i.e., migration of fibroblasts, re-epithelialization, angiogenesis and wound contraction. Chronic wounds are common and are considered a major public health problem. Therefore, there is the increasing need to discover new therapeutic strategies. MicroRNA (miRNA) research in the field of wound healing is in its early phase, but the knowledge of the recent discoveries is essential for developing effective therapies for the treatment of chronic wounds. In this review, we focused on recently discovered miRNAs which are involved in the proliferation phase of wound healing in the past few years and their role in wound healing.

## Wound Healing

Skin is the outer anatomical guard protecting us against the external environment. Skin is formed of 3 layers: epidermis, dermis and subcutaneous layer. Following skin injury, the synergetic interaction between different dermal cells in a process regulated at different levels is essential (Reinke and Sorg, 2012). The process of wound healing generally consists of four sequential but overlapping phases: (i) Hemostasis phase in which, platelets and coagulation factors lead to clotting and reduce blood loss (Kibe et al., 2017); (ii) Inflammation phase is characterized by infiltration of various inflammatory cells to the wound site to eliminate the pathogens and secrete cytokines and growth factors (Eming et al., 2014; Kibe et al., 2017); (iii) Proliferation phase is the rebuilding of extracellular matrix (ECM) at the wound site which involves angiogenesis, granulation tissue formation, collagen fibers deposition, epithelialisation and wound retraction (Young and McNaught, 2011); (iv) Remodeling phase is the maturation of scar tissue where type III collagen is replaced with type I collagen (Gurtner and Evans, 2000).

## Proliferation Phase of Wound Healing

Proliferation phase lasts from 3 days up to 2 weeks following skin injury. Collectively, the proliferative phase is achieved through three main steps: re-epithelialization, angiogenesis and the formation of granulation tissue.

### Re-epithelialization

In re-epithelialization, keratinocytes at the wound edges proliferate actively and migrate to re-establish coverage of the wound site (Cheng et al., 2016). In addition, stem cells from hair follicles or sweat glands in the vicinity of the wound also contribute to re-epithelialization (Miller et al., 1998; Roh and Lyle, 2006; Lau et al., 2009). Keratinocytes disengage the desmosome/hemidesmosome junctions and reorganize their cytoskeleton in order to migrate over the wound site (Cheng et al., 2016). Keratinocytes revert back to the normal phenotype after seizing migration and attach firmly to the re-established basement membrane and underlying dermis (Cheng et al., 2016).

### Angiogenesis

Angiogenesis is a complex process of growth of new vessels from pre-existing blood vessels. This process is initiated in the early healing process when the hemostatic plug has formed and as platelets release transforming growth factor (TGF)-β, platelet-derived growth factor (PDGF) and fibroblast growth factor (FGF) (Heun et al., 2017). Together with hypoxia-inducible factor-1 (HIF-1) and vascular endothelial growth factor (VEGF) which are released in response to hypoxia, these transcription factors and growth factors induce expression of several angiogenic genes, neovascularization and the repair of damaged blood vessels at the wound site (Young and McNaught, 2011; Heun et al., 2017). As angiogenesis proceeds, a rich vascular bed in the wound site that has many fold more capillaries than does normal tissue (DiPietro, 2016).

### Granulation Tissue Formation

After skin wounding, proliferation and migration of fibroblasts to the wound site are induced by growth factors released from the platelets (Young and McNaught, 2011). In addition, fibroblasts also derived from bone-marrow-derived mesenchymal stem cell (Sasaki et al., 2008). Fibroblasts secrete a loose matrix of ECM proteins (i.e., glycosaminoglycans, proteoglycans and hyaluronic acid), fibronectin and collagen (Singh et al., 2004; Young and McNaught, 2011). The resulting vascularized fibrous tissue replacing the hemostatic clot at the wound site is known as granulation tissue.

Following the formation of granulation tissue, fibroblasts change to α-smooth muscle actin-expressing myofibroblast phenotype, which is triggered by mechanical tension, focal adhesion protein Hic-5 and activated TGF-β (Hinz et al., 2001; Varney et al., 2016). Myofibroblast plays a major role in wound contraction, which begins approximately 7 days following injury, occurring at a rate of 0.75 mm/day (Young and McNaught, 2011). The final outcome will fill the wound gap with granulation tissue covered by surface epithelium, and the wound is ready for the last phase (remodeling).

## MicroRNA

MicroRNA (miRNA) is a small non-coding RNA molecule which consists of about 18–25 nucleotides in length (Parikh et al., 2014). The non-coding RNAs are RNA which do not code for proteins. However, some of them, including the ribosomal RNA, transfer RNA, small nuclear RNAs and small nucleolar RNAs, were reported to control various cell functions such as mRNA translation and regulation of gene expression (Mattick and Makunin, 2006) The non-coding RNA can be divided into short and long non-coding RNA depending on the number of nucleotides. Long non-coding RNAs are similar to mRNA and they range from 200 nucleotides to 100 kilobases in length. They are expressed in specific tissues to perform certain functions. Long non-coding RNAs include the antisense such as Oct4-pg5 and the brain-associated BC200 (Gibb et al., 2011). On the other hand, many researchers discovered different small non-coding regulatory RNAs (18–100 nucleotides in length) in both animals and plants which mainly include snoRNAs and miRNAs (Mattick and Makunin, 2006). miRNA as one of the small non-coding RNAs was discovered first in the early 1990s by Victor Ambros through studies with the lin-4 gene in the nematode *Caenorhabditis elegans*. In cells, miRNA plays a major role in the regulation of gene expression and controlling various processes such as metabolism, cell proliferation, differentiation and apoptosis (Gregersen et al., 2010). miRNA regulate gene expression through binding to the 3′ untranslated region (UTR) of mRNA. This binding leads to mRNA degradation which in turn leads to inhibition of protein translation (Nicoloso et al., 2009). Junk DNA was transcribed giving rise to different classes of non-coding RNA, including miRNAs, as a major part of human DNA which was thought to be with no function. Nowadays scientists have realized that a major part of the junk DNA is highly related to coding of miRNAs.

**FIGURE 1:**
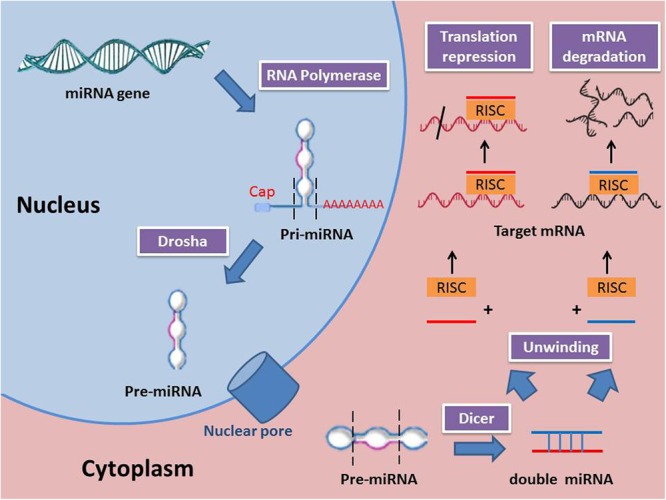
miRNA biogenesis and mechanisms of action (RISC, RNA-induced silencing complex; Pri-miRNA, Primary miRNA; Pre-miRNA, precursor miRNA).

miRNAs are synthesized from primary miRNAs (pri-miRNAs) in two stages by the action of two RNase III-type proteins: Drosha in the nucleus and Dicer in the cytoplasm (Kim et al., 2009). Genes for miRNAs are transcribed to primary miRNA (pri-miRNA), which is then processed within the nucleus to precursor miRNA (pre-miRNA) by a class 2 RNase III enzyme (Drosha). Next, the pre-miRNAs are transported from the nucleus to the cytoplasm where they are processed to become mature miRNAs by an RNase III-type protein (Dicer). miRNAs are attached to the Argonaute protein to produce the RNA-induced silencing complex (RISC) (Wahid et al., 2010). Several mechanisms were reported to regulate the biogenesis and expression of miRNAs (Kim, 2005). Those mechanisms include the single nucleotide polymorphisms (SNPs), which are induced by alternations in the nucleotides sequences of genes coding for miRNA. SNPs control the biogenesis and functions of miRNA by changing the coding sequences of variant miRNAs (Mishra et al., 2008). Moreover, RNA editing, one of the major pathways of RNA modification at the post-transcriptional level, can lead to different miRNA expression. For instance, almost 16% of pri-miRNAs synthesize is induced by A-to-I nucleotide editing (Kawahara et al., 2008). Some miRNA genes are affected by hypermethylation thus; nearly 10% miRNAs expression is regulated by DNA methylation. Meanwhile, inhibition of methylation resulted in a decreased in expression of certain miRNA (Han et al., 2007). The function of miRNAs is mainly related to the regulation of gene expression. A miRNA is complementary to a part of one or more mRNAs. Base pairing of RISC with the target RNA leads to inhibition of protein translation and/or mRNA degradation. miRNAs mainly bind their mRNA targets via a sequence between the 2nd and 8th nucleotides of their 5′ proximal region (Kawasaki and Taira, 2004). It is noteworthy to mention that miRNA interferes with the target gene expression through repression of mRNA translation or degradation of mRNA achieved by deadenylation from 3′ end and/or decapping from 5′ end. A previous study reported that 48% miRNA target genes are principally regulated by translation repression, 29% are controlled by mRNA degradation, and 23% are regulated by both mechanisms (Jin and Xiao, 2015). Moreover, in certain miRNA, the expression is promptly increased to millions of copies per cell aiming to degrade its target genes (Giraldez et al., 2006). In addition to the ability of miRNA to downregulate their target genes, they are also capable of upregulating the expression of genes. miRNA complex promotes the expression of target mRNAs similar to miRNA-mediated downregulation. The mRNA expression may be stimulated either by the direct effect of miRNA or indirectly through miRNA-mediated repression of other inhibitory miRNAs targeting the same gene (Valinezhad Orang et al., 2014). miRNA biogenesis and mechanism of action are shown in **Figure 1**.

## miRNA and Chronic Wound Healing

Chronic wounds such as leg ulcers, pressure ulcers, and diabetic foot ulcers (DFU) are common. Millions of people worldwide suffer from chronic wounds and their complications and thus are considered a major public health problem. In Germany, an epidemiology study revealed that there were 786,407 prevalent and 196,602 incident chronic wounds in the year of 2012 (Heyer et al., 2016). Nearly 3 million US citizens are suffering from pressure ulcer, while much more are suffering from venous ulcers (Shilo et al., 2007). It was also estimated that 15% of diabetic patients will suffer from diabetic ulcers, which is considered as a major source of morbidity and the main cause of hospitalization in diabetic patients (Aalaa et al., 2012; Leone et al., 2012; Shahbazian et al., 2013). Treating these chronic wounds is estimated to cost about $5–10 billion each year in the United States (Kuehn, 2007). In Singapore, the annual direct cost of chronic wound management in a tertiary hospital was about SGD 4.59 million while the total indirect costs (i.e., income loss due to hospitalization stay amounted to SGD 0.86 million (Tan et al., 2016).

Several studies have investigated the role of miRNA in skin metabolism. The following miRNAs were found to be heavily expressed in the skin: miR-152, miR-143, miR-126, miR-21, miR-27a, miR-214, miR-16, miR-203, miR-125b, miR-34a, miR-205, miR-27b, miR-30b, miR-125a, miR-191 and miR-200 family (Yi et al., 2006). Numerous studies have investigated the expression levels of various miRNAs in different phases of wound healing; suggesting miRNAs have a potential role in wound healing. In this review, we focus on the role of miRNA in the 3 main processes in the proliferation phase of wound healing: re-epithelialization, angiogenesis, and granulation tissue formation.

### Role of miRNA in Re-epithelialization

The re-epithelialization process is considered a crucial step in wound healing (Serarslan et al., 2007). Collectively, it involves migration of keratinocytes to cover the newly formed granulation tissue. Several studies have investigated the role of miRNA in regulating the re-epithelialization process (**Table 1**).

**Table 1:** miRNAs involved in the re-epithelialization process in wound healing.

miRNA	Effect	Target	Reference
miR-21	Enhances keratinocyte migration.	TIMP3, TIAM1	Yang et al., 2011
miR-31	Promotes keratinocyte proliferation and migration.	EMP-1	Li D. et al., 2015
miR-203	Inhibits keratinocyte proliferation and migration.	RAN, RAPH1, P63	Viticchie et al., 2012; Deppe et al., 2016
miR-204	Inhibits epithelial cells proliferation and migration.	SMAD4, SIRT1	Wang et al., 2013c; An et al., 2015
miR-205	Enhances epithelial cells proliferation and migration.	SHIP2	Yu et al., 2010
miR-210	Inhibits proliferation of epithelial cells.	E2F3	Giannakakis et al., 2008; Biswas et al., 2010

#### miR-21

miR-21 has been reported that miRNA plays a crucial role in cell proliferation, EMT and migration through silencing its target genes (Gabriely et al., 2008; Krichevsky and Gabriely, 2009). miR-21 promotes migration of cancer cells by targeting Reversion Inducing Cysteine-Rich Protein with Kazal motifs (RECK), TIMP metallopeptidase inhibitor 3 (TIMP3), and T-Cell Lymphoma Invasion And Metastasis 1 (TIAM1) genes (Gabriely et al., 2008; Cottonham et al., 2010). Recent studies demonstrated that miR-21 was highly expressed in the epidermis and hair follicle (Yi et al., 2006). Over-expression of miR-21 enhanced keratinocyte migration while miR-21 knockdown led to a remarkable delay in the re-epithelialization process (Yang et al., 2011). This may suggest that miR-21 is crucial for keratinocyte migration and re-epithelialization. TIMP3 and TIAM1 were identified as the direct target genes of miR-21 indicating that these genes are related to keratinocyte migration (Yang et al., 2011).

#### miR-31

miR-31 was reported to have a role in cell differentiation and hair growth (Mardaryev et al., 2010; Peng et al., 2012). Furthermore, the up-regulation of miR-31 has been reported in several skin conditions characterized by excessive keratinocyte proliferation such as psoriasis (Morhenn et al., 2013; Xu et al., 2013) and squamous cell carcinoma (Bruegger et al., 2013; Wang A. et al., 2014). The expression of miR-31 was gradually up-regulated in keratinocytes of wound edges in both inflammation and proliferative phases of wound healing. miR-31 promoted keratinocytes proliferation and migration by blocking epithelial membrane protein 1 (Li D. et al., 2015). Moreover, miR-31 expression was stimulated by Transforming Growth Factor Beta 2 (TGF-β2), which was significantly expressed in the wound area (Li D. et al., 2015).

#### miR-203

miR-203 is considered the most abundant keratinocyte-related miRNA in the epidermis (Sonkoly et al., 2007). Down-regulation of miRNA-203 promotes epithelial-mesenchymal transition (EMT) in various cancers (Wellner et al., 2009; Saini et al., 2011). miR-203 possesses an anti-proliferative activity through targeting the mRNA of the transcription factor p63 (Lena et al., 2008; Yi et al., 2008). P63 was proven to have a role in keratinocyte proliferation in the normal and diseased skin (Senoo et al., 2007; Lena et al., 2012). Two new targets of miR-203, Ras-related nuclear protein (RAN) and Ras Association and Pleckstrin Homology Domains 1 (RAPH1) are also found to be essential for the re-epithelialization process (Viticchie et al., 2012). miR-203 may control the expression of target proteins that are responsible for keratinocytes proliferation and migration. miR-203 was proven along with miR-210 to be key regulators of keratinocyte proliferation and migration (Deppe et al., 2016). Following injury, miR-203 expression was down-regulated in highly proliferating keratinocytes. Injections of antagomiR-203 in the dorsal skin of newborn mice increased the expression of its target mRNAs: RAN and RAPH1 (Viticchie et al., 2012). Inhibitors of both miR-203 and miR-210 could represent a potential therapy to enhance the re-epithelialization process.

#### miR-204

miR-204 is highly expressed in the epithelium of different eye tissues including the cornea, lens, and retina (Ryan et al., 2006; Wang et al., 2010). It has been reported that miR-204 is crucial for the development of both lens and retinal (Conte et al., 2010). Moreover, miR-204 is essential for the homeostasis of retinal pigment epithelium (Wang et al., 2010). Although miR-204 is heavily detected in the cornea, its expression was down-regulation during corneal wound healing. Increased expression levels of miR-204 lead to inhibition of cell proliferation and migration (An et al., 2015). Therefore, down-regulation of miRNA-204 may enhance epithelial wound healing. A few target genes of miRNA-204 have been reported such as SMAD Family Member 4 (SMAD4) and Sirtuin 1 (Wang et al., 2013a; Zhang et al., 2013). Furthermore, SIRT1 is an essential gene target of miR-204 in both corneal cell culture and mice corneal wound healing (An et al., 2015). In a recent study, it was observed that SIRT1 enhanced proliferation and migration epithelial cells (Wang et al., 2013c). This may support the fact that targeting of SIRT1 and SMAD4 by miR-204 leads to inhibition of proliferation and migration epithelial cells.

#### miR-205

Keratinocyte-specific miR-205 is expressed abundantly in epithelial tissues, where it can be detected in the basal and superficial layers of stratified squamous epithelium (Ryan et al., 2006; Yi et al., 2006). This indicates that miR-205 may be essential for development and homeostasis of epithelial cells (Gregory et al., 2008). It has been reported that miR-205 suppressed SH2-containing phosphoinositide 5-phosphatase 2 (SHIP2) in human epidermal keratinocytes and corneal epithelial keratinocytes cell lines (Yu et al., 2008). SHIP2 is a lipid phosphatase enzyme that dephosphorylates phosphatidylinositol 3,4,5-triphosphate (PIP3), a critical second messenger in various pathways including Akt and phosphoinositide-dependent kinase-1 which is essential for keratinocytes migration (Aman et al., 1998; Cantley, 2002). miR-205 plays a major role in keratinocyte migration thus, re-epithelialization process. miR-205 expression is down-regulated in migrating epithelial tongue during wound healing (Wang et al., 2016). This was proved by a study which used antagomiR-205 to reduce the expression levels of miR-205 in human epithelial cell lines. Treatment of human epithelial cell lines with antagomiR-205 increased SHIP2 levels and decreased the ability of the cells to close a scratch wounds (Yu et al., 2010). In addition, topical inhibition of miR-205 by administrating pluronic gel containing antagomiR-205 promotes keratinocyte migration in mouse wound model *in vivo* (Wang et al., 2016). Down-regulation of miR-205 resulted in an increased phosphorylation of the actin-severing protein cofilin, and a corresponding diminution of filamentous actin which suppressed the keratinocytes migration (Yu et al., 2010).

#### miR-210

Over-expression of miR-210 is a consistent feature of the hypoxia response (Corn, 2008; Huang and Zuo, 2014). Seven signature miRNAs (including miR-210) were found to be associated with low oxygen tension (Blick et al., 2015). HIF-1α is a transcription factor which regulates the hypoxic cycle of the cell has been found to arrest cell proliferation of keratinocytes (Scortegagna et al., 2009). Recent studies observed that HIF-1α may also serve as a transcription factor for the expression of miRNAs (Sen et al., 2009). Another study observed that ischemic wounds possessed increased expression levels of the HIF-1α-dependent miR-210 (Biswas et al., 2010). Furthermore, expression of E2F transcription factor 3 (E2F3) gene, a target gene of miR-210), which is essential for epithelial cells proliferation was significantly lower in ischemic wounds (Giannakakis et al., 2008). These observations clarify that miR-210 may inhibit the proliferation of epithelial cells in chronic wounds.

### Role of miRNA in Angiogenesis Process

Angiogenesis process is extremely vital for wound healing as it provides the essential oxygen and nutrients necessary for healing process (Li et al., 2003). Chronic wounds are characterized by depressed angiogenesis which is mainly attributed to the impairment of migration of the endothelial cells through blood vessel wall to form vascular sprouts and reduced level of VEGF in diabetic wounds (Brem and Tomic-Canic, 2007; Gallagher et al., 2007; Quattrini et al., 2008). miRNAs involved in angiogenesis are summarized in **Table 2**.

**Table 2:** miRNAs involved in the angiogenesis process of wound healing.

miRNA	Effect	Target	Reference
miR-1	Inhibits tube formation and endothelial cells proliferation.	VEGF-A	Xie et al., 2017
miR-21	Inhibits of proliferation and migration of endothelial cells.	PTEN, SMAD7	Jin et al., 2013; Jiang et al., 2015
miR-23a	Increases vascular permeability and cellular migration.	PHD1, 2	Hsu et al., 2017
miR-29b	Inhibits neovascularization.	VEGF, STAT3	Li et al., 2017b,c
miR-126	Enhances migration and repair of endothelial cells.	SPRED1	Cao et al., 2017
miR-133a/b	Stimulates proliferation and branch formation of endothelial cells.	TGF-β1	Sun et al., 2017
miR-146a	Enhances new blood vessels formation.	VEGF, Pak1	Seo et al., 2017
miR-210	Increases proliferation, migration and tube formation of endothelial cells.	Efna3	Hu et al., 2010; Wang et al., 2017
miR-218	Inhibits neovascularization.	ROBO1	Zhang X. et al., 2017
miR-377	Inhibits angiogenesis.	CD133, VEGF	Li et al., 2017a,b
miR-939	Disrupts vascular integrity and inhibits angiogenesis.	γ-catenin	Hou et al., 2017
miR-4530	Promotes angiogenesis.	VASH1	Zhang T. et al., 2017

#### miR-1

VEGF-A is considered the primary gene regulating angiogenesis process (Maeda et al., 1996). In human cancers, VEGF-A levels are usually associated with bad prognosis and high mortality rate (Juttner et al., 2006; Suzuki et al., 2010). miR-1 was found to suppress VEGF-A leading to inhibition of angiogenesis in tumor tissue (Stahlhut et al., 2012). The expression of miR-1 was frequently down-regulated in tumor tissues compared to corresponding normal tissues. Meanwhile, up-regulation of miR-1 remarkably inhibited the tube formation and endothelial cells proliferation by suppressing the expression of VEGF-A (Xie et al., 2017).

#### miR-21

miR-21 was reported to be over-expressed in multiple tumors in the last decade (Cheng and Zhang, 2010; Li et al., 2012). Moreover, recent studies showed that miR-21 is considered as a potent anti-angiogenic factor through the inhibition of proliferation and migration of endothelial cells (Jin et al., 2013; Jiang et al., 2015). miR-21 directly down-regulated the expression of both Phosphatase and Tensin Homolog (PTEN) and SMAD7 genes which are crucial in the regulation of angiogenesis process. Thus, over-expression of miR-21 inhibited endothelial cells proliferation, migration and tube formation (Wang et al., 2017).

#### miR-23a

miR-23a is expressed in many cancers including breast cancer and colon cancer as a key regulator of cellular apoptosis and proliferation (Li et al., 2013; Lian et al., 2013). A study showed that miR-23a was up-regulated in cells of lung cancer under hypoxic conditions leading to blocking its target prolyl hydroxylase 1 and 2 (PHD1 and 2) genes. Blocking of PHD1 and 2 led to the accumulation of HIF-1α in endothelial cells which in turn stimulated angiogenesis. Furthermore, miR-23a increased vascular permeability and cellular migration. On the other hand, down-regulation of miR-23a reduced angiogenesis process in cancer cells (Hsu et al., 2017).

#### miR-29b

miR-29b is a member of miR-29 family members which are known for their ability to induce transcriptional regulation, epigenetic modification and cell apoptosis (Han et al., 2010; Nguyen et al., 2011). Expression of miR-29b is weak in malignant cells as they possess anti-tumor functions. miR-29b is currently identified as a fundamental regulator of EMT which is considered a common pathway of cancer metastasis and chemotherapy resistance (Yan et al., 2015). A recent study has investigated the role of miR-29b in angiogenesis of breast cancer. The results showed that miR-29b was down-regulated in tumor tissues (Li et al., 2017b). Moreover, miR-29b showed anti-angiogenesis and anti-tumorigenesis activities by suppressing vascularization of cancer tissue. It was suggested that miR-29b targeted Akt3 pathway and inhibited Akt3 mediated VEGF (Li et al., 2017b). These results may support the role miR-29b as an inhibitor of angiogenesis process. Another study reported that up-regulation of miR-29b suppressed the angiogenesis of cervical cancer cells *in vitro* and inhibited neovascularization *in vivo* through targeting Signal Transducer and Activator of Transcription 3 (STAT3) gene. On the other hand, down-regulation of miRNA-29b could enhance angiogenesis of cancer cells (Li et al., 2017c).

#### miR-126

miR-126 plays a role in the vascular protection of endothelial cells (Zernecke et al., 2009). miR-126 can act as a tumor suppressor factor in the case of acute myeloid leukemia or act as an oncogene in case of colon cancer (Meister and Schmidt, 2010). miRNA-126 may support cancer progression through the promotion of angiogenesis process (Meister and Schmidt, 2010). It has been demonstrated that cigarette smoking released endothelial cells microparticles (EMPs) that contain miR-126. The increased miRNA-126-enriched EMPs promoted angiogenesis and endothelial homeostasis through enhancing migration and repair of endothelial cells (Cao et al., 2017). Sprouty Related EVH1 Domain Containing 1 (SPRED1) mRNA was identified as a gene target for miR-126. The miR-126 expression level in the peripheral blood of diabetic patients with DFU was lower than diabetic patients without DFU and the non-diabetic population (Zhang J. et al., 2017). Following maggot debridement therapy, the miR-126 expression was increased in diabetic patients with DFU (Zhang J. et al., 2017). The use of exosomes derived from miR-126-3p over-expressed synovial mesenchymal stem cells encapsulated in hydrogels in diabetic wound model successfully promote re-epithelialization and accelerate angiogenesis (Li et al., 2016).

#### miR-133a/b

In the human genome, there are three identified miRNA-133 species: miR-133a-1, miR-133a-2, and miR-133b found on chromosomes 18, 20 and 6 respectively (Ivey et al., 2008). TGF-β1 gene is considered as one of the major targets of miR-133 (Shan et al., 2009). miR-133a and miR-133b expression were significantly decreased in lung tissue after hyperoxia. It was observed that transfection of lung cells with miR-133a/b mimics restored cell proliferation and branch formation of endothelial cells. Moreover, miR-133a/b mimics stimulated expression of angiogenic factors in endothelial cells (Sun et al., 2017). The previous observations suggest that decreased expression of miR-133 inhibits angiogenesis process.

#### miR-146a

miR-146a is a well-identified miRNA which regulates inflammation and proliferation processes (Sonkoly et al., 2008). It has been reported that miR-146a was up-regulated in osteoarthritis (Yamasaki et al., 2009) and breast cancer (Lanczky et al., 2016). miR-146a targets P21 Activated Kinase 1 (PAK)1 gene, which in turn stimulated VEGF expression and formation of vascular branches. Moreover, miRNA-146a reduced fibrosis and enhanced new blood vessels formation (Seo et al., 2017). This suggests that miRNA-146 could regulate VEGF expression and promote angiogenesis process.

#### miR-210

miR-210 has been reported to be commonly up-regulated in cells of the ischemic heart and cardiac tumors (Devlin et al., 2011). Recent studies proved that over-expression of miR-210 stimulated angiogenesis and inhibited apoptosis of cardiac cells in case of myocardial infarction (Hu et al., 2010). Another study showed that miR-210 remarkably enhanced angiogenesis and cardiac function in ischemic heart disease (Wang et al., 2017). miR-210 was found to be up-regulated in mesenchymal stem cells which were used for treating myocardial infarction model. Moreover, miR-210 increased the proliferation, migration and tube formation of endothelial cells. Ephrin A3 (EFNA3) gene was identified as the target gene for miRNA-210 (Wang et al., 2017). The previously mentioned data elaborates the role of miR-210 as an angiogenic factor.

#### miR-218

miR-218 is well-known to be expressed by motor neurons and its down-regulation can result in hyperexcitability and neurodegeneration due to its potential role in homeostasis of the nervous system (Amin et al., 2015). Furthermore, miR-218 was identified in cancer cells such as squamous cell carcinoma (Uesugi et al., 2011). miR-218 was found to have a potential anti-tumor function in bladder cancer (Tatarano et al., 2011) and nasopharyngeal carcinoma (Alajez et al., 2011). A recent study reported that miR-218 inhibited neovascularization process *in vitro*. It was suggested that mechanism of action of miR-218 was mediated by targeting Roundabout Guidance Receptor 1 (ROBO1) gene (Zhang X. et al., 2017). Based on previous results, we may consider miR-218 as an anti-angiogenic therapy in case of cancers. However, miR-218 inhibitor could promote angiogenesis process.

#### miR-377

miR-377 is a crucial miRNA that plays a critical role in the pathogenesis of certain diseases such as diabetic nephropathy (Wang et al., 2008). miR-377 was down-regulated in specific cancers including ovarian cancer, prostatic cancer and lymphoma (Zhang et al., 2008; Arribas et al., 2013). Moreover, miR-377 was proven to inhibit proliferation and invasion of some human cancers such as glioblastoma (Zhang et al., 2014). Both i*n vitro* and *in vivo* study showed that miR-377 over-expression inhibited the growth and angiogenesis of esophageal cancer while its down-regulation showed opposite effects (Li et al., 2017a).

#### miR-939

miR-939 is a recently identified miRNA which was found to promote the cell proliferation in ovarian cancer (Ying et al., 2015). However, in breast cancer, miR-939 inhibited cell proliferation and metastasis (Di Modica et al., 2017). These findings may predict the variant roles of miRNA in different cancers. A recent study revealed that miRNA-939 was down-regulated in ischemic heart disease patients and it was found to disrupt vascular integrity and inhibit angiogenesis through targeting γ-catenin gene (Hou et al., 2017).

#### miR-4530

miR-4530 expression was down-regulated in patients with diabetic retinopathy. Interestingly, miR-4530 possessed the ability to stimulate angiogenesis in endothelial cells (Ding et al., 2016). Vasohibin-1 (VASH1) gene was identified as one of the targets of miRNA-4530 (Zhang T. et al., 2017). VASH1 gene is over-expressed in endothelial cells leading to inhibition of angiogenesis process as a negative feedback (Watanabe et al., 2004; Kimura et al., 2009). Thus, by suppressing VASH1, miR-4530 may promote angiogenesis. miR-4530 was demonstrated to promote angiogenesis in breast cancer and to suppress breast cancer by inhibiting cell proliferation and induction of apoptosis (Zhang T. et al., 2017).

### Role of miRNA in Granulation Tissue Formation

The formation of the granulation tissue is a significant step in wound healing as it regulates and organizes the growth of the wound tissue (Asahara et al., 1999; Schultz and Wysocki, 2009). In this phase, granulation tissue formed is composed of different molecules including glycosaminoglycans, proteoglycans, and hyaluronic acid. Numerous miRNAs have been reported to support the formation of granulation tissues (**Table 3**).

**Table 3:** miRNAs involved in granulation tissue formation in wound healing.

miRNA	Effect	Target	Reference
miR-29	Inhibits collagen synthesis and angiogenesis.	HSP47	Guo et al., 2017
miR-98	Decreases viability and increases apoptosis of fibroblasts.	Col1α1	Bi et al., 2017
miR-141-3p	Inhibits cell proliferation, migration of fibroblasts and enhances cell apoptosis.	–	Feng et al., 2017
miR-185	Inhibits growth of fibroblasts.	TGF-β1, Col-1	Xiao et al., 2017

#### miR-29

miR-29 was reported to have a significant role in the regulation of some biological processes including cell apoptosis (Kole et al., 2011) and proliferation (Wei et al., 2013). Various studies identified multiple target genes for miR-29 including genes coding for ECM component such as collagen and elastin genes (Kriegel et al., 2012; Wang et al., 2013b). The miR-29b expression was significantly decreased during the healing process while the heat shock protein 47 (HSP47, a post-transcriptional target of miR-29b,) level was markedly increased, following full-thickness excisional wound and burn wound models (Zhu et al., 2016). The local administration of miR-29b lentivirus inhibits HSP47 expression, collagen synthesis, and angiogenesis during skin wound healing (Zhu et al., 2016). In addition, miR-29b reduces excessive scar formation by inhibition of TGF-β1/Smad/CTGF signaling pathway (Guo et al., 2017).

#### miR-98

miR-98 has a role in the development of variant cancers. It inhibits tumor angiogenesis and invasion by decreasing the matrix metalloproteinase-11 enzyme levels (Siragam et al., 2012). A recent study showed that miRNA-98 can regulate collagen, type I alpha 1 (Col1α1) gene expression in skin fibroblasts. Following transfection of fibroblasts with miR-98 mimic, there was a remarkable decline in Col1α1 gene expression leading to declining in the cell viability and increased apoptosis. On the other hand, transfection of cells with miR-98 inhibitor resulted in opposite effects (Bi et al., 2017). Fibroblast apoptosis is known as a key regulator in the development of normal and pathological scar (Scharstuhl et al., 2009). So that, miR-98 may play a role in wound healing by enhancing fibroblast proliferation and controlling scar formation.

#### miR-141-3p

miR-141-3p was reported to suppress the growth and metastasis of prostatic tumor (Liu et al., 2017). Similarly, miR-141-3p inhibited invasion and metastasis of glioma (Peng et al., 2016). The over-expression of miR-141-3p inhibited cell proliferation and migration of skin fibroblasts as well as enhanced cell apoptosis, while its down-regulation resulted in the opposite effects (Feng et al., 2017).

#### miR-185

miR-185 located on chromosome 22, was reported in various types of cancer including lung cancer (Li S. et al., 2015), hepatoma (Qadir et al., 2014), and stomach cancer (Tan et al., 2014). miR-185 has a role in wound healing and fibroblast migration (Yang and Yee, 2014). A recent study suggested that over-expression of miR-185 inhibited the growth of fibroblasts, through direct targeting of TGF-β1 and Col-1 genes, both serve a critical role in the development of hypertrophic scarring (Xiao et al., 2017).

## Therapeutic Strategies for Wound Healing and Future Directions

miRNA-based therapy will be a revolution in the advanced therapy for chronic wounds as they will be able to modulate a group of genes through the use of a single miRNA. The base upon which therapeutic miRNAs will be used relays on the ability to reduce the damaging miRNAs and up-regulate the levels of the beneficial miRNAs which could be achieved by several methods. Up-regulation of potentially beneficial miRNAs can be achieved by using synthetic double-stranded oligonucleotides called miRNA mimics. Mimics are double-stranded RNA which consists of the same sequence of the target miRNA in one strand which will be incorporated into the RISC complex (Fasanaro et al., 2010). The down-regulation of potentially harmful miRNAs can be achieved by complementary oligonucleotides antagomiRs which act as competitive inhibitors through binding to the mature miRNA or lead to inhibition of miRNA maturation by binding to the pre-miRNA. Successful delivery of either mimics or antagomiRs depends mainly on their resistance to destruction in different tissues and binding affinity to the target miRNA. Therefore, chemical modifications of the oligonucleotides are required. Collectively, miRNA regulators can be delivered to mammalian tissues through either intravenous injection of oligonucleotides or conjugation of those oligonucleotides with other lipophilic molecules as high-density lipoproteins to facilitate their uptake by cells (Fasanaro et al., 2010). Further studies investigating the role of different miRNAs in the phases of wound healing along with screening their target genes are recommended. A better understanding of the molecular mechanism of wound healing may provide better treatment and advanced solutions to chronic wounds. Various miRNA delivery systems were discovered in the last decade to convey miRNAs of choice into cells of wound tissue aiming to promote wound healing. Among those delivery systems, viral vectors are considered the most common and effective, despite few disadvantages including mutagenesis and restricted capacity for genetic material. Meanwhile, non-viral miRNA delivery systems, such as cationic polymers, peptides, and liposomes, are recognized by their lower toxicity, water solubility, and resistance to endonucleases or phagocytosis. These vectors are loaded with miRNAs and transfected into cutaneous cells to induce their function (Meng et al., 2017). Researchers conducted several experiments to deliver certain beneficial miRNAs into wound tissue. A previous study revealed significant effects of antihypoxamiR encapsulated lipid nanoparticles on ischemia during wound healing (Ghatak et al., 2016). Others reported that delivery of miR-27b mimics may improve wound healing through stimulating the bone marrow-derived angiogenic cell function in patients with type 2 diabetes mellitus (Wang J.M. et al., 2014). Systematic injection of anti-miR-26a improved angiogenesis process in mice through increasing SMAD1 gene expression (Icli et al., 2013).

## Conclusion

Numerous studies have demonstrated the important role of miRNA in wound healing, especially in chronic wounds. miRNAs are regulatory molecules that contribute to numerous aspects and phases of wound healing. They may play an important role to accelerate the wound healing process in chronic wounds.

## Author Contributions

Conceptual framework and design: NAG and SD. Searched reference and drafted the article: AS and ST. Critically revised the article: NAG and SD. All authors reviewed and accepted the final version of the article.

## Conflict of Interest Statement

The authors declare that the research was conducted in the absence of any commercial or financial relationships that could be construed as a potential conflict of interest.
